# Neuroimaging Findings on Amodal Completion: A Review

**DOI:** 10.1177/2041669519840047

**Published:** 2019-04-08

**Authors:** Jordy Thielen, Sander E. Bosch, Tessa M. van Leeuwen, Marcel A. J. van Gerven, Rob van Lier

**Affiliations:** Radboud University, Donders Institute for Brain, Cognition and Behaviour, Nijmegen, the Netherlands

**Keywords:** amodal completion, occlusion, functional neuroimaging, EEG, fMRI, MEG, SUR

## Abstract

Amodal completion is the phenomenon of perceiving completed objects even though physically they are partially occluded. In this review, we provide an extensive overview of the results obtained from a variety of neuroimaging studies on the neural correlates of amodal completion. We discuss whether low-level and high-level cortical areas are implicated in amodal completion; provide an overview of how amodal completion unfolds over time while dissociating feedforward, recurrent, and feedback processes; and discuss how amodal completion is represented at the neuronal level. The involvement of low-level visual areas such as V1 and V2 is not yet clear, while several high-level structures such as the lateral occipital complex and fusiform face area seem invariant to occlusion of objects and faces, respectively, and several motor areas seem to code for object permanence. The variety of results on the timing of amodal completion hints to a mixture of feedforward, recurrent, and feedback processes. We discuss whether the invisible parts of the occluded object are represented as if they were visible, contrary to a high-level representation. While plenty of questions on amodal completion remain, this review presents an overview of the neuroimaging findings reported to date, summarizes several insights from computational models, and connects research of other perceptual completion processes such as modal completion. In all, it is suggested that amodal completion is the solution to deal with various types of incomplete retinal information, and highly depends on stimulus complexity and saliency, and therefore also give rise to a variety of observed neural patterns.

## Introduction

We live in a complex world full of objects that are (partly) hidden by other objects. In fact, in natural situations, we encounter many more partially occluded or temporarily hidden objects than fully visible objects. Therefore, the bottom-up input to our visual system is incomplete and fragmented. Nevertheless, we do not perceive these occluded objects as incomplete, fragmented, and unrelated, but rather as complete, consistent, coherent, and whole objects. Strikingly, we seem to be unaware of this fragmented reality surrounding us and take for granted the completed reality that our brain creates. Somehow, the brain is capable of constructing a completed representation of incomplete retinal images. This inverse problem is ill-posed due to its incompleteness and therefore subject to an infinite amount of possible completions. Still, our brain fills in the incomplete parts of occluded objects effortlessly and does so within a split second.

The process of completing objects in the absence of direct visual sensory input due to occlusion is called *amodal completion* (Michotte & Burke, 1951; Michotte, Thinès, & Crabbé, 1964; Michotte, Thinès, Costall, & Butterworth, 1991; for a review, see R. van Lier & Gerbino, 2015). The term *amodal* refers to the fact that the occluded parts are not subjectively visualized (i.e., are not represented in a sensory modality). Specifically, one does not see the occluded parts of an occluded object, but one does appreciate that it continues behind the occluder. The term *completion* indicates that the occluded parts are somehow represented, despite their physical absence. In other words, the initially incomplete representation is extended to a completed representation, which contains filled-in information at the area of occlusion. The concept of amodal completion can be somewhat confusing because there is no visual sensation of the (completed) occluded parts. So what is completed, or what does this completion entail? Also, the degree of detail of the completion may vary between different completions. For example, a partly occluded straight contour might have a more pronounced phenomenological presence than for instance a partly occluded human face. In the latter case, *amodal presence* would perhaps be a better term. Regardless, in this review, we continue to use the term amodal completion for all cases in which image parts are occluded.

Amodal completion is not to be confused with *modal completion*, which is another type of perceptual completion. Modal completion involves the vivid visual perception of illusory contours and surfaces (Kanizsa, 1976, 1985). Specifically, within modal completion an object seems floating in front of another object in the absence of direct visual sensory input. Even though the illusory figure is physically indistinguishable from its background, the phenomenological experience is as if its contours seem brighter than the background.

**Figure 1.**
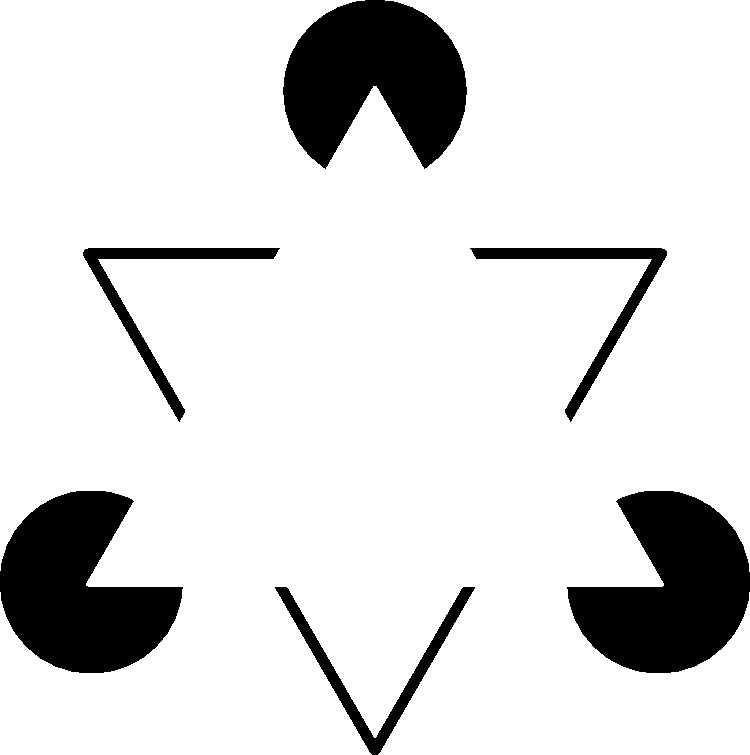
The Kanizsa triangle. The physical arrangement of three filled-in black circles with cut-out parts and three line-drawing black arrow-heads on an equiluminant black background creates the subjective experience of a modally completed triangle pointing up and an amodally completed triangle pointing down. Adapted from Kanizsa (1976).

The famous Kanizsa triangle, shown in Figure 1, is an appropriate illustration to explicate the differences between amodal and modal completion. Physically, the only objects present in the Kanizsa triangle are three black discs each with a cut-out triangular part and three black arrow-heads, all presented on an equiluminant white background. Perceptually, the discs together form an upward pointing triangle in the near depth plane (i.e., modal completion) and the arrow-heads together form a downward pointing triangle in the far depth plane (i.e., amodal completion). Note that the modally completed triangle occludes not only the amodally completed triangle but also the three black discs, which are not perceived as the so-called Pac-Men but as amodally completed whole discs.

Amodal completion might involve an initial stage which holds the representation of the physical object only (i.e., a mosaic-stage that is not yet completed) and a second stage at which the occluded object is completed (i.e., a completion stage where the whole object is represented). Behavioral studies suggest that the completion process unfolds over time rapidly, namely within 200 to 400 ms in pictorial displays (Bruno, Bertamini, & Domini, 1997; Sekuler & Palmer, 1992) and already within 100 ms if objects are perceived stereoscopically (Bruno et al., 1997). Under certain conditions, amodal completion is cognitively impenetrable, which is for instance evident from magical tricks relying on the amodal presence or the amodal absence of objects (Ekroll, Sayim, & Wagemans, 2017; Ekroll & Wagemans, 2016). In addition, already 3.5- to 4.5-month-old infants are capable of amodal completion (e.g., de Wit, Bauer, Oostenveld, Fries, & van Lier, 2008; Kellman & Spelke, 1983), object permanence (e.g., Baillargeon, 1987), and volume completion (e.g., Soska, Adolph, & Johnson, 2010; Vrins, Hunnius, & van Lier, 2011). These observations together highlight the automatic nature of amodal completion.

Amodal completion can be guided by local cues such as continuations of contours at T-junctions (von Helmholtz & Southall, 1924), linear continuations (Wouterlood & Boselie, 1992), inflected curved contours (Takeichi, Nakazawa, Murakami, & Shimojo, 1995), curved continuation by the relatability criterion (Kellman & Shipley, 1991), or vector field combinations (Fantoni & Gerbino, 2003). On the contrary, completions based on global cues depend on shape regularities like symmetry (Buffart, Leeuwenberg, & Restle, 1981; R. van Lier, van der Helm, & Leeuwenberg, 1994; R. J. van Lier, van der Helm, & Leeuwenberg, 1995; R. J. van Lier, Leeuwenberg, & van der Helm, 1995). An account for such global regularities is provided by the structural information theory (Leeuwenberg, 1969, 1971; van der Helm & Leeuwenberg, 1991, 1996). At later stages of amodal completion, clear top-down influences might mediate the completion process. For instance, object knowledge (Hazenberg & van Lier, 2016; Vrins, de Wit, & van Lier, 2009; Yun, Hazenberg, & van Lier, 2018), object familiarity (Hazenberg, Jongsma, Koning, & van Lier, 2014), surrounding objects (Rauschenberger, Peterson, Mosca, & Bruno, 2004), and preceding objects (Plomp & Van Leeuwen, 2006; Zemel, Behrmann, Mozer, & Bavelier, 2002) have been shown to have contextual effects on amodal completion. These observations emphasize the influence of top-down processes on amodal completion.

In the current review, we focused on the *neural mechanisms* behind amodal completion. We considered studies for this review based on three criteria. First, the study had to be involved in neuroimaging, meaning that some measure of brain activity had to be recorded and analyzed. Second, whenever the stimulus set contained objects that were occluded by other objects, then the study would classify as a candidate amodal completion study. Both static (i.e., nonmoving) occlusion as well as dynamic occlusion were considered. We also included studies in which objects were gradually occluded until they were completely invisible. With this requirement, we did not consider studies that used stimulus omissions like cut-out parts of objects without any physical or phenomenological experience of occlusion (e.g., Morgan, Petro, & Muckli, 2016; Smith & Muckli, 2010), nor studies that blurred images and investigated effects of image deterioration on object recognition (e.g., H. Tang et al., 2014). Third, the occluding object (i.e., the occluder) had to be physically distinct (e.g., in terms of color) from the occluded object or the background. With this requirement, we did not consider studies on modal completion (e.g., Peterhans & von der Heydt, 1989). In the “Discussion” section, however, we briefly discuss similarities and differences regarding neural mechanisms between amodal and modal completion and also consider perceptual completion under other image distortions like cut-out pieces and blurring.

The main aim of this review is to provide a clear and thorough summary of the neural mechanisms of amodal completion as reported throughout the literature so far (summarized in Table 1). In addition, we provide an overview of insights from computational models of amodal completion, and we relate amodal completion to other perceptual completion processes such as modal completion. The main body of this review is organized around three major questions. First, *where* are amodally completed objects represented? For instance, do both low-level areas and high-level areas represent the completed object or do low-level visual areas only represent the object that is physically presented? Note that we do not assume one area dedicated to amodal completion but instead discuss at what level of the visual hierarchy the amodal completion is represented. Second, *when* is amodal completion achieved? Specifically, what are the temporal dynamics of amodal completion and at which stage of the visual hierarchy is amodal completion achieved? Furthermore, does amodal completion involve a purely feedforward process or is it interleaved with recurrent and feedback processes? Note that we do not assume one time point at which amodal completion is resolved but instead discuss what type of temporal dynamics are involved. And third, *how* is the amodal completion neurally represented? Specifically, are the invisible parts of the occluded object represented as if these parts were visible (i.e., without occlusion), or is there only a high level awareness of the occluded parts?

**Table 1.** Literature Overview.

Study	Subjects	Method	Display	Occlusion	Studied areas
Assad and Maunsell (1995)	M = 3	SUR	Pictorial	Dynamic	pPC
Kovács et al. (1995)	M = 2	SUR	Pictorial	Dynamic	ITC
Caputo et al. (1999)	H = 7	EEG	Pictorial	Static	Occipital
Sugita (1999)	M = 2	SUR	Stereoscopic	Dynamic	V1
Zhou et al. (2000)	M = 4	SUR	Pictorial	Static	V1, V2, V4
Bakin et al. (2000)	M = 2	SUR	Stereoscopic	Static	V1, V2
Lee and Nguyen (2001)	M = 2	SUR	Pictorial	Static	V1, V2
Baker et al. (2001)	M = 2	SUR	Stereoscopic	Dynamic	STS
Umilta et al. (2001)	M = 2	SUR	Stereoscopic	Dynamic	PMC
Lerner et al. (2002)	H = 11	fMRI	Pictorial	Static	V4, LOC
Yin et al. (2002)	H = 8	fMRI	Pictorial	Dynamic	V1, V2, LOC, MT
Olson et al. (2003)	H = 9 + 4	fMRI	Pictorial	Dynamic	V1, V2, MT
Lerner et al. (2004)	H = 10 + 7	fMRI	Pictorial	Static	V1, V2, V3, V4, LOC
Murray et al. (2004)	H = 9	EEG	Pictorial	Static	Occipital, posterior parietal
Johnson and Olshausen (2005)	H = 40 + 12 + 12	EEG	Pictorial	Static	Occipital, posterior parietal
Rauschenberger et al. (2006)	H = 10	fMRI	Pictorial	Static	V1, V2, V4, LOC
Liu et al. (2006)	H = 10	MEG	Pictorial	Static	Occipital, temporal, parietal
Plomp et al. (2006)	H = 10	MEG	Pictorial	Static	Occipital, temporal, parietal
de Wit et al. (2006)	H = 9	MEG	Pictorial	Static	Occipital, temporal
Hulme and Zeki (2007)	H = 13	fMRI	Pictorial	Dynamic	V1, V2, LOC, FFA, PMC, SFG, PFC
Shuwairi et al. (2007)	H = 10	fMRI	Pictorial	Dynamic	Full brain
Weigelt et al. (2007)	H = 10	fMRI	Pictorial	Static	V1, V2, V3, ITC
Harris and Aguirre (2008)	H = 10 + 13 + 7	fMRI	Stereoscopic	Static	FFA
Hegdé et al. (2008)	H = 12	fMRI	Stereoscopic	Static	DF, LOC
Makin et al. (2009)	H = 17	EEG	Pictorial	Dynamic	Occipito-parietal
Chen et al. (2009)	H = 12	EEG	Stereoscopic	Static	Frontal, parietal, occipital
Chen et al. (2010)	H = 8	fMRI	Stereoscopic	Static	V1, V2, FFA
Bushnell et al. (2011)	M = 2	SUR	Pictorial	Static	V4
Ban et al. (2013)	H = 8 + 8	fMRI	Pictorial	Dynamic	V1, V2, V3
Hazenberg et al. (2014)	H = 12	EEG	Pictorial	Static	Frontal, central
Kosai et al. (2014)	M = 2	SUR	Pictorial	Static	V4
Hazenberg and van Lier (2016)	H = 28	EEG	Pictorial	Static	Occipital, parietal
Fyall et al. (2017)	M = 2	SUR	Pictorial	Static	V4, PFC
Erlikhman and Caplovitz (2017)	H = 10	fMRI	Pictorial	Dynamic	V1, V2, V3, FFA, LOC, MT
Chen et al. (2017)	H = 17	EEG	Pictorial	Static	Occipital, posterior parietal
de Haas and Schwarzkopf (2018)	H = 7	fMRI	Pictorial	Dynamic	V1, V2, V3
Rajaei et al. (2018)	H = 15	MEG	Pictorial	Static	Posterior temporal

The major methodological aspects of the neuroimaging studies on amodal completion. Subjects are denoted as M (monkeys) and H (humans), and numbers denote the sample size in individual neuroimaging experiments. EEG = electroencephalography; fMRI = functional magnetic resonance imaging; MEG = magnetoencephalography; SUR = single-unit recording. Abbreviations of studied areas: DF = dorsal foci; FFA = fusiform face area; ITC = inferior temporal cortex; LOC = lateral occipital complex; MT = middle temporal; pPC = posterior parietal cortex; PFC = prefrontal cortex; PMC = premotor cortex; SFG = superior frontal gyrus; STS = superior temporal sulcus.

## Methodological Aspects

Before discussing the findings of neural mechanisms involved in amodal completion, it is important to discuss the different methodological approaches that are used throughout the literature to study the neural mechanisms of amodal completion. Here, we discuss the data acquisition protocols, stimulus conditions, stimulus displays, and stimulus types as used throughout this literature. These diverging approaches can of course influence findings and may account for differences in the reported results. An overview of the major design details are given in Table 1.

### Data Acquisition

Data acquisition protocols can have a substantial impact on what can be interpreted from the data. Several techniques have been used to study neural mechanisms of amodal completion including single-unit recordings (SURs) in awake monkeys (Assad & Maunsell, 1995; Baker, Keysers, Jellema, Wicker, & Perrett, 2001; Bakin, Nakayama, & Gilbert, 2000; Bushnell, Harding, Kosai, & Pasupathy, 2011; Fyall, El-Shamayleh, Choi, Shea-Brown, & Pasupathy, 2017; Kosai, El-Shamayleh, Fyall, & Pasupathy, 2014; Kovács, Vogels, & Orban, 1995; Lee & Nguyen, 2001; Sugita, 1999; Umilta et al., 2001; H. Zhou, Friedman, & von der Heydt, 2000), electroencephalography (EEG) with humans (Caputo, Romani, Callieco, Gaspari, & Cosi, 1999; J. Chen, Liu, Chen, & Fang, 2009; S. Chen, Töllner, Müller, & Conci, 2017; Hazenberg et al., 2014; Hazenberg & van Lier, 2016; Johnson & Olshausen, 2005; Makin, Poliakoff, & El-Deredy, 2009; Murray, Foxe, Javitt, & Foxe, 2004), magnetoencephalography (MEG) with humans (de Wit, Bauer, Oostenveld, Fries, & van Lier, 2006; Liu, Plomp, van Leeuwen, & Ioannides, 2006; Plomp, Liu, van Leeuwen, & Ioannides, 2006; Rajaei, Mohsenzadeh, Ebrahimpour, & Khaligh-Razavi, 2018), and functional magnetic resonance imaging (fMRI) in humans (Ban et al., 2013; J. Chen, Zhou, Yang, & Fang, 2010; de Haas & Schwarzkopf, 2018; Erlikhman & Caplovitz, 2017; Harris & Aguirre, 2008; Hegdé, Fang, Murray, & Kersten, 2008; Hulme & Zeki, 2007; Lerner, Hendler, & Malach, 2002; Lerner, Harel, & Malach, 2004; Olson, Gatenby, Leung, Skudlarski, & Gore, 2003; Rauschenberger, Liu, Slotnick, & Yantis, 2006; Shuwairi, Curtis, & Johnson, 2007; Weigelt, Singer, & Muckli, 2007; Yin, Shimojo, Moore, & Engel, 2002).

These data acquisition methods differ predominantly in their temporal and spatial resolution. Specifically, a method with a high temporal resolution can indicate more precisely *when* a measured phenomenon happened, while a method with high spatial resolution can denote more precisely *where* an effect is located. Ideally, the best way forward would be to use a method that has both a high temporal resolution as well as a high spatial resolution. SUR provides such a method as it operates on individual neurons and records with high sampling rates. However, SUR is applied only at local scales and therefore does not cover large brain areas. In addition, SUR is highly invasive as it requires surgery to insert electrodes in the brain and is thus typically done with primates instead of humans. Unfortunately, the currently available noninvasive neuroimaging techniques either allow a high temporal resolution at the cost of a low spatial resolution (EEG, MEG) or allow a high spatial resolution at the cost of a low temporal resolution (fMRI).

It should be noted here that several studies did aim to localize the source of activity within EEG, which is possible with source localization algorithms (e.g., Murray et al., 2004). Also, several studies attempted to find temporal patterns using fMRI, for instance using a backward masking paradigm (e.g., Lerner et al., 2004; Rajaei et al., 2018).

### Stimulus Conditions

**Figure 2.**
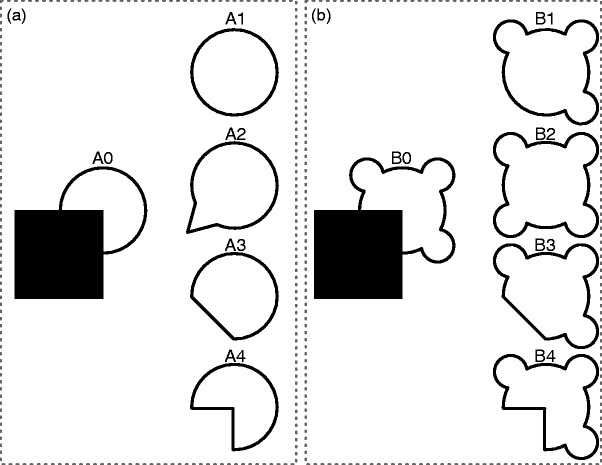
Stimulus conditions and completions. A0 comprises a convergent occlusion stimulus where local and global completion tendencies converge to the same completion (A1). Note that completion A1 results from a simple curvilinear continuation of the partly occluded contours (i.e., local completion), while the resulting shape is also highly regular (i.e., global completion). Completions A2 and A3 and the mosaic interpretation A4 appear rather anomalous. B0 comprises a divergent occlusion stimulus in which local and global tendencies diverge toward different completions. Completion B1 again is the result of a simple curvilinear continuation of the partly occluded contours (i.e., local completion), whereas completion B2 maximally accounts for global regularity (i.e., global completion). Completions B3 and the mosaic interpretation B4 appear rather anomalous. Adapted from Sekuler (1994).

To study amodal completion, preferably three stimulus conditions are required. Throughout this review, we refer to these three conditions as *occluded, completed*, and *mosaic*. However, do note that the way these conditions are implemented throughout literature might differ substantially and not all studies comprise all three conditions, let alone that they are named this particular way. Figure 2 illustrates these conditions as part of both convergent shapes where both local and global cues trigger the same completion (Figure 2(a)) as well as divergent shapes where local and global cues predict different completions (Figure 2(b)).

In the occluded condition, an object is occluded by another object (Figure 2(A0) and (B0)). This condition naturally forms the main condition in which amodal completion is studied. Stimuli used for the occlusion condition vary from partially occluded objects to fully occluded (i.e., invisible) objects, from static occlusion to dynamic occlusion, and from pictorial displays to stereoscopically presented displays. It should be noted that these choices may have a substantial effect on which cortical areas are involved. For instance, during dynamic full occlusion working memory and motor areas might be recruited, which is not the case in static partial occlusion.

The completed condition forms one extreme of the possible interpretations of the occlusion condition (Figure 2(A1), (B1), and (B2)). The completed condition in fact acts as a baseline supposing what the representation would look when amodal completion occurred to its full extent. In other words, if the occluded object would be completed in full detail, then its representation should be similar to the one of the object when it is fully visible (i.e., without occluder). In general, the completed condition presents objects in its full detail either without or in front of the occluding object.

The mosaic condition represents the other extreme of possible interpretations of the occlusion condition (Figure 2(A4) and (B4)). The mosaic condition also acts as a baseline but supposes that no amodal completion occurred at all. This would mean that only the physical parts of the object would be represented (i.e., the occlusion area cut out from the object). Stimuli used for the mosaic condition vary from occluded but scrambled displays, to physical gaps between the occluded object and its occluder, to disappearing objects compared to gradually occluded objects.

### Stimulus Display and Occlusion Types

For amodal completion to happen stimuli have to be displayed in such a way that occlusion is perceived. Throughout the body of literature occlusion was triggered by using pictorial displays or by stereoscopically presented displays. The former uses simple 2D stimulus configurations while the latter presents distinct images to individual eyes to manipulate depth perception (e.g., with red-green anaglyph images). Pictorial displays utilize only monocular depth cues (e.g., T-junctions) while stereoscopic displays take advantage of binocular depth cues (e.g., binocular disparity). This may affect the strength of the occlusion display and hence the salience of the representation of the amodally completed object.

In addition, the occlusion can either remain static (i.e., an occlusion pattern is directly shown) or can proceed dynamically (i.e., objects are visible at first and gradually move behind an occluder after which they reappear again). It should be clear that the former method involves more automatic processes involved in amodal completion, while the latter more likely involves visual working memory as well, because the object was first perceived in full vision. Furthermore, (visual) motion areas might be recruited during dynamic occlusion because of movement of the occluder or occluded object.

Most studies aimed at investigating the neural mechanisms of amodal completion have used static displays presented pictorially (Bakin et al., 2000; Bushnell et al., 2011; Caputo et al., 1999; S. Chen et al., 2017; de Wit et al., 2006; Fyall et al., 2017; Hazenberg et al., 2014; Johnson & Olshausen, 2005; Kosai et al., 2014; Lee & Nguyen, 2001; Lerner et al., 2002, 2004; Liu et al., 2006; Murray et al., 2004; Plomp et al., 2006; Rauschenberger et al., 2006; Weigelt et al., 2007). Apart from these studies, few have used stereoscopic depth cues to manipulate the depth planes of the statically presented stimuli. Some have done so by varying the disparity of the occluder (J. Chen et al., 2009, 2010; Harris & Aguirre, 2008), while others varied the disparity of the object, while keeping the occluder at zero disparity (Hegdé et al., 2008).

Alternatively, several studies investigated dynamic occlusion of pictorial images, for instance objects that get gradually occluded (Hulme & Zeki, 2007; Kovács et al., 1995; Yin et al., 2002), or contrary, the objects themselves that move gradually behind a static occluder (Assad & Maunsell, 1995; Ban et al., 2013; de Haas & Schwarzkopf, 2018; Erlikhman & Caplovitz, 2017; Makin et al., 2009; Olson et al., 2003; Shuwairi et al., 2007).

Yet others have studied dynamic object occlusion using stereoscopic stimuli. Either a dynamic object moved along a disparity manipulated occluder (Sugita, 1999) or 3D objects passed behind a static occluder (Baker et al., 2001; Umilta et al., 2001).

**Figure 3.**
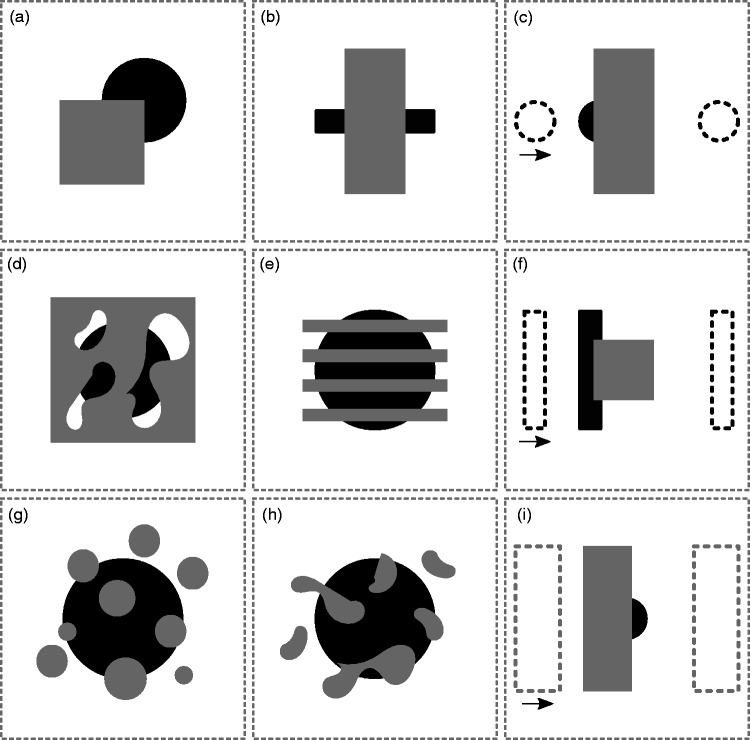
Stimulus displays and occlusion types. As used throughout the amodal completion neuroimaging studies, (a), (b), (d), (e), (g), and (h) depict static occlusion displays, whereas (c), (f), and (i) show dynamic occlusion. In (c) and (f), the occluded object moves, whereas in (i) the occluder moves. Note that the stimulus types as used here, i.e., circular and rectangular shapes, are for illustrative purpose only. Throughout literature, other stimulus types have been used.

An illustrative overview of all occlusion paradigms that have been used throughout the neuroimaging studies of amodal completion is shown in Figure 3. The left two columns in Figure 3 show static occlusion displays, while the right column shows dynamic stimuli. Note that, in Figure 3, we abstracted from differences in pictorial or stereoscopic displays as well as stimulus types. To summarize, static partial occlusions (Figure 3(a)) were studied most (Bushnell et al., 2011; S. Chen et al., 2017; de Wit et al., 2006; Hazenberg et al., 2014; Lee & Nguyen, 2001; Liu et al., 2006; Murray et al., 2004; Plomp et al., 2006; Rauschenberger et al., 2006; Weigelt et al., 2007; H. Zhou et al., 2000), static occlusions where two parts need to be connected (Figure 3(b)) were studied (Bakin et al., 2000; Hazenberg & van Lier, 2016; Sugita, 1999), displays with a large static occluder with wholes (Figure 3(d)) were studied to a lesser extent (J. Chen et al., 2009, 2010; Hegdé et al., 2008), objects occluded by a static grid (Figure 3(e)) were investigated by a few (Caputo et al., 1999; Harris & Aguirre, 2008; Lerner et al., 2002, 2004), objects occluded by other scattered circular objects (Figure 3(g)) were studied (Fyall et al., 2017; Kosai et al., 2014; Rajaei et al., 2018), but also similarly using other occluding objects (Figure 3(h)) (Johnson & Olshausen, 2005; Kovács et al., 1995), and dynamic full occlusion (Figure 3(c)) was studied (Assad & Maunsell, 1995; Erlikhman & Caplovitz, 2017; Makin et al., 2009; Olson et al., 2003; Shuwairi et al., 2007; Umilta et al., 2001), dynamic partial occlusion (Figure 3(f)) (Ban et al., 2013; de Haas & Schwarzkopf, 2018), and dynamic occlusion where the occluder was moving (Figure 3(i)) was studied (Hulme & Zeki, 2007; Yin et al., 2002).

### Stimulus Types

Throughout the literature on neural mechanisms of amodal completion, several simple stimuli have been employed, such as oriented bars (Bakin et al., 2000; Caputo et al., 1999; de Haas & Schwarzkopf, 2018; Sugita, 1999), squares, circles, crosses, and stars (Assad & Maunsell, 1995; Ban et al., 2013; Bushnell et al., 2011; S. Chen et al., 2017; de Wit et al., 2006; Erlikhman & Caplovitz, 2017; Fyall et al., 2017; Hazenberg et al., 2014; Kosai et al., 2014; Kovács et al., 1995; Liu et al., 2006; Plomp et al., 2006; Rauschenberger et al., 2006; Weigelt et al., 2007), Kanizsa figures (Lee & Nguyen, 2001; Murray et al., 2004), or moving balls (Makin et al., 2009; Olson et al., 2003; Shuwairi et al., 2007).

Alternatively, studies presented images of more complex natural objects such as animals, tools, vases, and food (Hazenberg & van Lier, 2016; Hegdé et al., 2008; Johnson & Olshausen, 2005; Lerner et al., 2002, 2004; Rajaei et al., 2018; Yin et al., 2002), houses (Hulme & Zeki, 2007), faces (J. Chen et al., 2009, 2010; Harris & Aguirre, 2008; Hulme & Zeki, 2007), hand movements executed by the experimenter (Umilta et al., 2001), or gradual occlusion of the experimenter (Baker et al., 2001).

Simple stimuli such as oriented bars might restrict the neural representation only to low-level visual areas, whereas more natural scenes and actual objects such as tools, faces, and houses might recruit higher order visual areas.

## Where Is It Represented?

Here, we discuss which cortical areas are implicated in the process of amodal completion. Specifically, which areas represent the occlusion condition in a similar way as the completion condition and therefore can be categorized as involved in amodal completion? Conversely, which areas show a correspondence between the occlusion condition and the mosaic condition and can therefore be categorized to be not involved in amodal completion or at least represent the mosaic stage in the process? An in-between option might exist too, where areas represent the occlusion condition in between the completed condition and mosaic condition. This might suggest that these areas are involved in amodal completion but represent an occluded object weaker than when it is fully visible. Note, however, that areas that show a correspondence between the completed and occluded condition might be involved in representing the completed object, but this does not imply that such areas are involved in the completion process itself. Also, we do not assume one specific spatial pattern or brain area for amodal completion, but rather ask the question whether low-level and high-level areas are involved in the process, and under which conditions the occlusion condition yields similar response patterns as the completed condition.

### Low-Level Visual Areas

Evidence for amodal completion manifested in early visual cortex comes predominantly from SURs in monkeys. It was first shown using stereoscopically presented bars, where V1 neurons responded only to an oriented bar when an occluder was placed at uncrossed disparity (i.e., in the near depth plane) so that it seemed occluded (Sugita, 1999). These V1 neurons responded similarly to an occluded bar as to a fully visible bar, suggesting V1 neurons represent an occluded object in its fully completed form. Later, these results were confirmed using the flank facilitation effect and stereoscopic displays (Bakin et al., 2000). In 9% of V1 and 42% of V2 neurons, responses were largest when bars were occluded, compared to when they were intersected. H. Zhou et al. (2000) found neurons in V1, V2, and V4 to code for border-ownership, and observed these neurons to respond to an edge only when it was at a certain side of an object (i.e., belonging to the occluded or occluding object).

V4 is a likely candidate for object completion because of its shape-selective responses. Indeed, Bushnell et al. (2011) showed that individual neurons from monkey V4 showed strong responsiveness to specific sharp convexities. However, the response readily decreased when these sharp convexities were placed in context with an occluding object. Another study showed that such shape-selectivity of monkey V4 neurons decreased when more occlusion was applied (Kosai et al., 2014). Finally, two transient response peaks were found in monkey V4 neurons, where the second peak was mediated by higher visual areas to facilitate recognition under occlusion (Fyall et al., 2017).

Within human neuroimaging studies, the involvement of low-level visual areas seems more obscured. Using Kanizsa figures (Lee & Nguyen, 2001) and dynamic occlusion (Ban et al., 2013; Erlikhman & Caplovitz, 2017), V1-3 showed larger activity in the occlusion condition than in the mosaic condition, providing evidence for amodal completion in early visual areas. Another study attempted to fit a population receptive field (pRF) model using amodally completed oriented bars (de Haas & Schwarzkopf, 2018). The authors showed that there was a significant correlation between the parameters of a pRF model in the completed condition and the parameters of a pRF model in the occlusion condition. However, activity was larger in the completed than in the occluded condition in V1, V2, and V3. Also Hulme and Zeki (2007) showed that V1 and V2 activity was larger to faces and houses in the completed than in the occluded condition. However, the authors argued that such contrast may reflect the obvious higher spatial detail on the screen in the completed as compared to occluded condition.

Using an fMRI adaptation paradigm and short (100 ms) and long (250 ms) presentation durations, V1 and V2 were shown to represent the mosaic at short presentation durations and the completion at longer presentation durations (Rauschenberger et al., 2006). This might suggest that V1 and V2 are definitely involved in the representation of the amodally completed object but that this process takes time to evolve. However, in a study with a similar adaptation paradigm this effect was not replicated (Weigelt et al., 2007). Instead, V1, V2, and V3 were shown to represent the mosaic, not the completion at 300 ms. In accordance, Olson et al. (2003) showed that dynamic occlusion of a ball revealed no different activity in V1 and V2 than an abruptly disappearing ball during the occlusion period. In line with this, V1, V2, V3, V4, and V8 showed no difference between activity in the occlusion condition and the mosaic condition (Lerner et al., 2002, 2004). Finally, Erlikhman and Caplovitz (2017) attempted to decode object identity during dynamic occlusion. They observed significant decoding from early visual areas V1 and V2 before the occlusion period (i.e., when the object was still visible), but found no significant decoding when the object was occluded (i.e., when it was behind the occluder).

In summary, the most compelling evidence for the involvement of early visual cortex in amodal completion comes from SURs in monkeys using simple stimuli like oriented bars. However, within human neuroimaging studies, only little evidence has been found in favor of amodal completion in early visual areas. In these studies, although stronger activity was found in the occlusion condition than in the mosaic condition, also weaker responses were found in the occlusion condition than in the completed condition. This might suggest amodal completion in early visual cortex, but the representation of an amodally completed object is weaker than a representation of a fully visible object. However, several other studies showed that the response in the occluded condition was similar to the one observed in the mosaic condition, suggesting that completion is not represented in early visual areas at all.

### High-Level Visual Areas

The lateral occipital complex (LOC) has been repeatedly reported throughout literature as a likely candidate to be involved in amodal completion or to be a region in which amodal completion has been established. LOC has been reported to be involved in the recognition of objects (Grill-Spector, Kourtzi, & Kanwisher, 2001) and is shown to be invariant to low-level detail like contour, while maintaining its sensitivity to shape (Kourtzi & Kanwisher, 2001). LOC was also found to be invariant to occlusion, expressed by statistically indifferent responses in LOC to the completed and occluded shapes (Rauschenberger et al., 2006) and houses (Hulme & Zeki, 2007). In addition, one study showed significant decoding of the identity of an occluded object from LOC activity patterns (Erlikhman & Caplovitz, 2017). However, several studies have also found LOC activity to be larger in the completed condition than in the occluded condition (Hegdé et al., 2008; Lerner et al., 2002, 2004; Yin et al., 2002). In line with this, an fMRI study showed a decreased LOC amplitude when more distortion was applied to slit-viewed objects (Yin et al., 2002).

Several studies investigated occluded faces for which the fusiform face area (FFA) has been observed to be highly specialized (Kanwisher & Yovel, 2006). Like LOC, FFA was shown to be invariant to occlusion, yielding similar responses to complete faces as to occluded faces (Hulme & Zeki, 2007). These results were confirmed with stereoscopically presented faces, although only for longer presentation times (250 or 350 ms) and not for shorter ones (50 or 150 ms) (J. Chen et al., 2010). Contrary to this, an earlier study failed to find completion effects of faces in FFA (Harris & Aguirre, 2008).

Similar effects were found in the inferior temporal (IT) cortex, which is a large region including LOC and FFA and therefore implicated in the recognition of a broad range of objects. Kovács et al. (1995) observed larger ITC responses in the completed condition than in the occluded condition, and the response amplitude decreased when more occlusion was applied. Using an fMRI adaptation paradigm, ITC showed more adaptation to the completed object than to the mosaic one, which suggests ITC represents the completed object (Weigelt et al., 2007).

Studies using dynamic occlusion paradigms reported effects of occlusion also in (visual) motor areas. Activity was reported to be the same in the complete condition and the occlusion condition in middle temporal (MT) (Yin et al., 2002) as well as premotor cortex (PMC; Umilta et al., 2001). In line with these observations, larger activity was found in MT in the occluded condition than in the mosaic condition (Olson et al., 2003). In one study, even larger MT activity was reported to occluded than to completed faces and houses (Hulme & Zeki, 2007).

Several other areas have been implicated in amodal completion. One of these were the dorsal foci, the dorsal stream of the visual pathway, which showed larger responses in the occluded than in the completed condition and larger responses in the completed than in the mosaic condition (Hegdé et al., 2008). Larger responses in the completed than in the occluded condition were also reported for both posterior parietal cortex (Assad & Maunsell, 1995) and inferior parietal cortex (Olson et al., 2003). Neurons in the superior temporal sulcus (STS) were shown to start responding from the onset of occlusion reaching a peak between 1 and 4 seconds after full occlusion, which could maintain even up to 11 seconds after full occlusion (Baker et al., 2001). In addition, the posterior frontal gyrus was shown to respond more in the completed than in the occluded and more in the occluded than in the mosaic condition (Lerner et al., 2002, 2004). Finally, preferential responses to occluded objects were observed in the superior frontal gyrus (Hulme & Zeki, 2007) and prefrontal cortex (Fyall et al., 2017; Hulme & Zeki, 2007).

In summary, LOC seems to be a core area involved in the representation of global objects, notwithstanding their physical incompleteness. FFA seems to be the LOC counterpart specific to faces showing an invariance to occlusion for faces specifically. Both LOC and FFA seem to represent an occluded object to the same extent as a fully visible object, although a few studies reported weaker responses to occluded objects than to the completed ones. In all, we can conclude that LOC and FFA are definitely involved in the representation of occluded objects, but might do so to a weaker extent than when the objects are fully visible.

Other candidate regions are those that are involved in the representation of dynamically occluded objects. Motor areas like MT and PMC seem to respond even more to dynamically occluded objects than to fully visible objects. These (visual) motor areas might be implicated in object permanence as these were also observed to maintain activity for object presence when lights were turned off (Graziano, Hu, & Gross, 1997). Also, several higher level areas across the temporal, parietal, and frontal lobes were implicated during dynamic occlusion. These areas might be recruited because of the involvement of visual working memory and visual imagery in these paradigms.

## When Is It Represented?

Here, we provide an overview of when amodal completion is actually achieved. Specifically, when is the physically incomplete sensory input completed to a completed representation of the occluded object? Does a mosaic stage exist that is gradually completed along the visual hierarchy? Is amodal completion a purely stimulus-driven feedforward process or does it require recurrent and feedback processes, the latter triggered by knowledge and experience, to fill in the missing pieces of the occluded object? Again, we restrict ourselves here to the literature that explicitly measured neural activity. Also, we do not assume one specific temporal pattern for amodal completion in general, but rather ask the question whether amodal completion relies on feedforward processes only, and under which conditions recurrent and feedback processes are required.

### Feedforward

As discussed in the previous section, individual V1 neurons from monkeys responded to occluded oriented bars. These V1 responses to occluded bars had a similar latency as those to visible bars, both of 80 to 100 ms (Sugita, 1999). This would suggest a feedforward approach that is solved already at the level of primary visual cortex. Lateral connections might be used to sense outside the classical receptive field (Sugita, 1999).

In contrast, Lee and Nguyen (2001) found responses in the occlusion condition to be 55 ms later than in the completed condition. Also IT neurons were observed to respond after 158 ms in the occlusion condition, while IT neurons responded already at 108 ms in the completed condition (Kovács et al., 1995). STS neurons were not responsive as long as an object was still visible, but became increasingly more active during occlusion reaching peak activity 1 to 4 seconds after full occlusion (Baker et al., 2001). This response was also shown to persist as long as 1 to 11 seconds after complete occlusion. These observations might still suggest a feedforward process, but one that requires more time under occlusion.

In EEG and MEG studies, peak latencies at occipital recording locations were found at 129 ms (Johnson & Olshausen, 2005), 142 to 188 ms (Caputo et al., 1999), and 140 to 238 ms (Murray et al., 2004). An occipital N170 and frontal P190 at 131-221 ms were found to occluded faces (J. Chen et al., 2009). Completions that involved local completions were observed to resolve faster (123.9 ms) than those based on global completions (125.1 ms), while the physical object evoked activity already after 118.9 ms (Liu et al., 2006; Plomp et al., 2006). An occipital P1 at 115 to 140 ms and a N1 at 140 to 170 ms were found depending mostly on completions defined by structural information, while a P3 was found at 300 to 400 ms depending on both structural completions as well as completions guided by knowledge (Hazenberg & van Lier, 2016). Also, a contralateral delay activity was observed at occipito-parietal sites at 500 to 1,200 ms after occlusion (S. Chen et al., 2017). de Wit et al. (2006) showed that the amplitude of the mismatch negativity (MMN) was weaker to local (i.e., completion based on linear extensions) and global completions (i.e., completions based on symmetry and repetition) than to anomalous completions of convergent shapes at both occipital (160–250 ms) as well as temporal (240–360 ms) regions (for examples of convergent and divergent shapes, see Figure 2). The MMN was also shown to be weaker for local completions than to global completions for divergent shapes at occipital (180–230 ms) and temporal (270–350 ms) regions.

Several attempts were made to investigate the temporal evolution of amodal completion with fMRI. This was done by either presenting the stimuli for varying durations or by using backward masks. J. Chen et al. (2010) found that V1 and V2 activities were larger in the mosaic than in the occluded condition at 50 and 150 ms. In contrast, FFA showed larger amplitudes in occluded than in the mosaic condition only at 250 and 350 ms (J. Chen et al., 2010). Also Rauschenberger et al. (2006) reported that at 100 ms, the mosaic was represented in both low-level and high-level areas, whereas at 250 ms, the completed object was represented. However, LOC was found to respond to occluded objects as it does to completed objects already at 60 ms, although amplitudes were larger when objects were presented for 250 ms (Lerner et al., 2004).

In summary, it can be noted that the abovementioned results vary substantially. Of course, this variability can be caused by differences in tasks, paradigms, and type of stimuli used.

### Feedback and Recurrent

Direct evidence for feedback processes in amodal completion has been reported throughout literature. First, Lee and Nguyen (2001) found that the latency of individual V2 neural activity to occluded objects was 30 ms before V1 activity. They additionally showed that occluded objects evoked responses 55 ms later than visible objects. This suggests both longer processing times for occluded objects than for visible ones and feedback connections from V2 to V1 involved in amodal completion. In addition, individual V4 neurons' shape selectivity was shown to be modulated by higher level areas, specifically ventrolateral prefrontal cortex (Fyall et al., 2017).

Implicit evidence for the existence of recurrent and feedback processes in amodal completion has been reported by two studies. Hazenberg et al. (2014) reported that learning specific object names could influence the recognition of predominantly divergent completion shapes that are more ambiguous because of diverging local and global completions. This was reflected in differential EEG P2 amplitudes that were significantly altered after learning as compared to before learning. In a subsequent EEG study, clear effects of structure (bottom-up) and knowledge (top-down) were shown, reflected by a P1 and N1 that were guided by structure only, and a P3 that was affected by both structure and knowledge (Hazenberg & van Lier, 2016).

Interestingly, an MEG decoding study showed that more recurrent connections were evident in the occluded than in the completed condition (Rajaei et al., 2018). The authors additionally showed that backward masking had an effect only on recognition in the occluded but not in the completed condition. Finally, they showed that a feedforward artificial neural network model did not explain their results, while a similar neural network with local recurrent connections did.

In summary, several studies reported direct evidence of recurrent and feedback connections at several levels of the visual hierarchy necessary for amodal completion. These observations are confirmed by EEG studies that showed clear effects of familiarity and learning on both behavioral as well as neural responses. Finally, a computational model incorporating recurrent connections better explained MEG data under occlusion than did a feedforward model. These results opt for feedback mechanisms involved in amodal completion.

## How Is It Represented?

Here, we address whether the representation of the invisible parts of an occluded object involves a detailed low-level representation as it would be when the object was not occluded or merely an abstract representation. Specifically, are the occluded parts completed in full detail as when it would be visibly presented, or is there only an tacit awareness created of the occluded parts of an object? Showing that the response patterns as observed in occluded conditions are similar to those found in completed conditions is not enough to answer this question. If similar spatial detail could be extracted from occluded regions as from visible regions, then it could be inferred that information is available about these features. Of course, low-level information might only be captured in low-level visual areas such as V1 because these have the typical small receptive field sizes required to represent an object in detail, while high-level areas that have larger receptive fields will have a more abstract representation by definition. Still, it remains a question of importance whether the representation of an occluded object is similar to the same object without occlusion.

A fruitful way to investigate how the completion is represented neurally is to attempt to decode low-level object information from its neural representation. An MEG study showed significant decoding of object category despite occlusion (Rajaei et al., 2018). Similarly, an fMRI study showed significant decoding of occluded object identity from higher level visual areas such as LOC (Erlikhman & Caplovitz, 2017). However, the same study showed that decoding was not possible from low-level visual areas even though responses in the occluded condition were stronger than those in the mosaic condition. Decoding of object identity from low-level areas was restored again when the object reappeared from behind its occluder (Erlikhman & Caplovitz, 2017).

Evidence converges to a weaker representation of the occluded object within low-level areas. Population receptive field (pRF) parameters correlated significantly between the completed and occlusion condition (de Haas & Schwarzkopf, 2018). However, activity was larger in the completed than in the occlusion condition in V1, V2, and V3. Several studies also found LOC activity to be larger in the completed than in the occluded condition (Hegdé et al., 2008; Lerner et al., 2002, 2004; Yin et al., 2002). In line with this, an fMRI study showed a decreased LOC amplitude when more distortion was applied to slit-viewed objects (Yin et al., 2002).

Several high-level influences might mediate these findings. It has been shown that the amount of attention mediates the occlusion effect (J. Chen et al., 2010). Specifically, with a simple task at fixation, the occlusion condition showed similar response patterns as the completed condition. However, with a demanding task that diverted attention away from the objects, the effect vanished. This suggests that amodal completion highly depends on the saliency of the occluded object.

In summary, low-level areas seem to represent the occluded object without detailed low-level properties. Instead, in higher level areas, the occluded object does seem to be represented in its completed form. However, one should be careful with this interpretation, as the information present in higher level areas might come from the visible parts of the occluded stimulus due to increasingly larger receptive fields further up the visual hierarchy. So, it remains rather uncertain to what extent the invisible parts of an occluded object are processed as if there was no occlusion in both low-level as well as high-level visual areas.

## Discussion

In this review, we have provided an overview of the neuroimaging literature on amodal completion. Table 1 shows all studies as included in this review. To our knowledge, this table includes all studies in the field of amodal completion in which an attempt was made to unravel the neural dynamics of amodal completion. With that in mind, we did not consider research into modal completion or other perceptual completion processes like perception under other challenging conditions, for example, cut-out pieces or blurring.

The debate on *where* amodal completion takes place or which cortical areas are involved in amodal completion still remains. Especially in early visual areas, amodal completion was predominantly found using simple bar-like stimuli and SURs in monkeys. Within human neuroimaging studies, only a few studies found activity related to amodal completion in early visual areas, while others found evidence for mosaic-like interpretations. These ambiguous results might be explained by the fact that simple stimuli that rely on linear continuations can be directly completed by interpolation, something that might be resolved already early on in the visual hierarchy. In contrast, more complex sceneries might depend more on the salience of the image and goal-oriented nature of the perceiver before they are filled-in and might require more high-level and recurrent and feedback processing. For instance, mental imagery might be required to make a more vivid visual representation, which is known to recruit neural circuits more overlapping with core visual perception (Dijkstra, Bosch, & van Gerven, 2017). In addition, more complex stimuli of course require more complex features to be processed and might therefore rely on more high-level visual areas to guide the completion process. In line with this, areas with larger receptive fields like LOC and FFA have been reported to represent occluded objects as their completed counterparts and thus seem invariant to occlusion. In all, this seems to suggest that amodal completion is not a unique singular phenomenon, but incorporates many levels of completions, depending on the specific stimulus properties. Simple objects might be already completed at low-level areas, while more complex objects require more downstream visual areas.

The debate on *when* an occluded object is completed also remains an open-ended question. From several studies, it can be concluded that amodal completion requires recurrent and feedback processing. Specifically, several studies found low-level areas to be active only after higher level areas were activated. Still, when such recurrence is really required might again depend on the type of stimuli used and the paradigm involved. For instance, the completion effects found for simple oriented bar stimuli in monkey early visual cortex might rely on close range or recurrent connections that perform linear interpolations (Sugita, 1999). In accordance, an MEG decoding study showed more recurrent processing for recognition under occlusion than for fully visible objects (Rajaei et al., 2018). However, when more complex stimuli are used, more feedback connections might be required to incorporate knowledge and experience from more downstream visual areas. The reliance on recurrent and feedback connections, however, might not directly imply that recognition of objects under occlusion is more involved in cognition rather than perception, since perception itself also requires recurrent and feedback processes. In sum, evidence seems to suggest that different levels of amodal completion require more or less recurrent and feedback processing over feedforward processing. This might depend on the complexity of the completion and thereby causes variation in the latencies for different types of stimuli and paradigms.

The debate on *how amodal completion* is represented neurally also remains unanswered. Several studies showed significant decoding, while others could not decode object identity under occlusion. This discrepancy is interesting, because one might expect that within amodal completion, at least in low-level visual areas, there is nothing completed, because there is no phenomenological visual experience of the occluded object and therefore no need to represent the low-level details of the occluded parts. However, we do seem to know more about the hidden parts of an occluded object than just its presence, which makes it intriguing how such awareness of object characteristics can unravel without subjective experience of its visual details. The current development of more sophisticated decoding techniques might provide the means to probe the representations as involved in amodal completion and might in turn reveal whether the invisible parts of an occluded object are actually completed, or whether we should better refer to the phenomenon with the term amodal presence instead of amodal completion.

In summary, the three main questions within this review remain unanswered and open for further debate and experimentation. From the body of literature in which brain activations have been measured when looking at (partly) occluded objects, we can propose a clear hypothesis which poses amodal completion as a collection of perceptual completion phenomena, highly dependent on properties of the occluded object like its complexity and saliency.

### Computational Models

Apart from directly studying how the brain copes with occluded objects, one might implement specific types of architectures for object perception in computational models and test hypotheses using simulations. There are several studies that modeled the processes as involved in amodal completion in such a computational way.

The neocognitron is one of the first feedforward artificial neural network models to perform visual pattern recognition (Fukushima, 1988; Fukushima & Miyake, 1982). It is inspired by the human brain and models lateral geniculate nucleus cells that perform contrast extracting operations, and simple and complex cells from early visual cortex that perform edge extracting operations. The neocognitron has also been shown to be able to perform recognition of occluded objects when it was extended with an additional layer that inhibits the neuronal activation evoked by irrelevant contours from the occluding object (Fukushima, 2001). Later, the neocognitron was extended with feedback processing to allow also the reconstruction or completion of occluded objects (Fukushima, 2005), which was later refined using V2-like bend extraction cells (Fukushima, 2010). This research line is interesting, as it suggests that recognition of partially occluded objects might suffice with a feedforward approach, but that filling in of the occluded parts demands feedback processes.

Other biologically inspired models have focused on interpolation and extrapolation processes using feedforward networks only and thereby contrast the intuition made earlier. A biologically inspired model for modal completion was already proposed (Heitger, Von Der Heydt, Peterhans, Rosenthaler, & Kübler, 1998). Based on local feedforward processes only, Kalar, Garrigan, Wickens, Hilger, and Kellman (2010) extended this model and showed that it could perform illusory as well as occluded contour completion. This notion nicely illustrates the identity hypothesis, which postulates that modal and amodal completion might be driven by similar mechanisms (Kellman & Shipley, 1991). However, Kalar et al. (2010) observed that the local aspect limited the capacity of the model, in need of global influences. Later, a feedforward model incorporating both local and global cues was found to be able to explain object segmentation under occlusion (Oliver, Haro, Dimiccoli, Mazin, & Ballester, 2016). In this model, the relatability account (Kellman & Shipley, 1991) was implemented to construct many possible completions of an occluded object, together with a Bayesian model that could select the most plausible completion based on its perceptual complexity (van der Helm, 2011; R. van Lier et al., 1994).

Apart from the more biologically plausible neural network models, state-of-the-art models from the deep learning community have also been used to study object recognition under occlusion. First, Spoerer, McClure, and Kriegeskorte (2017) showed the importance of lateral connections for object recognition under occlusion and also showed the importance of recurrent connections for object recognition under other challenging conditions like Gaussian additive noise. In any case, object recognition always gained accuracy from recurrent processing. In line with this, it has been shown that a state-of-the-art feedforward neural network could not detect occluded objects at human levels, but that this performance was restored when recurrent connections were added (Rajaei et al., 2018).

Apart from the literature on cognitive neuroimaging and computational neuroscience, a substantive body of literature deals with what is called image denoising and inpainting. The aim of image denoising and inpainting is to recover an image that is contaminated with noise, for instance occlusion patterns. For these types of operations, several models have been used like stacked sparse denoising autoencoder (SSDA) (Xie, Xu, & Chen, 2012), double channel SSDA (Cheng, Wang, Gong, & Hou, 2015), Markov random field theory (Z. Zhou, Wagner, Mobahi, Wright, & Ma, 2009), Boltzmann machine (Y. Tang, Salakhutdinov, & Hinton, 2012), and long short-term memory autoencoder and generative adversarial networks (Zhao, Feng, Zhao, Yang, & Yan, 2018). Also in these types of models, a common trick to deal with occlusion is to detect the occluding object in a separate circuitry (see e.g., Cheng et al., 2015; Zhao et al., 2018) like the inhibitory masking layer in Fukushima (2001) and to include recurrent processing (see e.g., Zhao et al., 2018).

### Other Perceptual Completion Processes

Here, we consider a few other completion processes, specifically modal completion and perceptual completions under image distortions like cut-out pieces and blurring. Given the phenomenological similarities and differences, it seems expedient to have a closer look at similarities and differences regarding their neurological backgrounds, and specifically their overlap with amodal completion.

Modal completion might overlap with amodal completion in terms of neural mechanisms, because both involve similar processes that segregate figure and ground, and both involve interpolation and extrapolation processes to infer physically absent contours and surfaces. Specifically, in modal completion illusory contours are completed, while in amodal completion occluded contours are completed. The hypothesis that both perceptual completion processes operate under a shared (neural) framework is called the *identity hypothesis* (Kellman & Shipley, 1991). This framework was also implemented in a computational model that is capable of interpolation and extrapolation of both occluded as well as illusory contours (Kalar et al., 2010). However, the identity hypothesis has been under debate (see e.g., Anderson, 2007; Kellman, Garrigan, Shipley, & Keane, 2007).

The overlap between amodal and modal completion also seems evident from the neuroimaging literature. For instance, similar to amodal completion, V1 and V2 neurons have been observed to respond to oriented illusory bars outside their classical receptive field, where 4% of V1 neurons and 32% of V2 neurons showed a reliable response to illusory contours (Peterhans & von der Heydt, 1989). Several studies also directly investigated the overlap between modal and amodal completion and found similarities (Bakin et al., 2000; H. Zhou et al., 2000), although amodal completion seemed to evoke a stronger EEG P3 response (Murray et al., 2004). In addition, larger responses were found to modally than to amodally completed objects in V1 and V2, but both were smaller than the visible object (Lee & Nguyen, 2001). Also, in two split-brain patients, modal completion recruited both hemispheres equally, while amodal completion recruited predominantly the right hemisphere (Corballis, Fendrich, Shapley, & Gazzaniga, 1999). Recurrent processing seems important for modal completion too. Using transcranial magnetic stimulation, feedback connections to V1 and V2 were shown to be necessary for modal completion (Wokke, Vandenbroucke, Scholte, & Lamme, 2013). For a review of the neural correlates of modal completion, see Seghier and Vuilleumier (2006).

Other situations in which perceptual completion is necessary are when images contain cut-out pieces or blurred parts. Such a perceptual completion process might involve the filling in of object parts that are not physically present, but in these cases in the absence of occlusion. Two studies showed the ability to decode the object identity from cut-out parts of an image (Morgan et al., 2016; Smith & Muckli, 2010). In both studies, decoding was driven by both V1 and V2, but predominantly V1. This is rather unexpected, because a cut-out part of a stimulus would correspond to the mosaic condition within amodal completion, in which no completion would be expected. In line with this, Johnson and Olshausen (2005) showed that such cut-out representation yields different EEG responses than the occluded counterpart. Apart from cut-out pieces, the effect of blurring on object recognition was also investigated and shown to rely mostly on recurrent processes (O'Reilly, Wyatte, Herd, Mingus, & Jilk, 2013; H. Tang et al., 2014; Wyatte, Curran, & O'Reilly, 2012; Wyatte, Herd, Mingus, & O'Reilly, 2012; Wyatte, Jilk, & O'Reilly, 2014).

### Final Remarks

Everything that we subjectively perceive is a creation of the brain. Perception is first guided by stimulus-driven feedforward processing, but it is also regulated by recurrent and feedback processes. In this context, the dissociation between perception and cognition becomes rather obscure, as the separation might not always be as clear as one would expect. Also in amodal completion, processes are predominantly initiated by bottom-up input but completed under top-down control. The exact neural mechanisms as implicated in amodal completion are just beginning to be unraveled. From this review, it seems evident that different levels of amodal completion exist that depend on the complexity and saliency of the occluded objects, and hence yield different observed neural patterns. Future research should investigate these possible different levels of amodal completion and how this influences which brain areas are involved and to what extent recurrent and feedback processing is required. Getting a solid understanding of the neural mechanisms behind amodal completion could in turn also provide crucial insights into its overlap with other perceptual phenomena such as modal completion, mental imagery, and visual working memory. In broader terms, a better understanding of amodal completion will also aid in elucidating how our complex visual system can build a stable and accurate representation of the visual world around us from incomplete retinal images. With that, the somewhat neglected area of amodal completion is in fact at the heart of visual processing, creating the rich environment we experience from moment to moment.
